# Racial/Ethnic Disparities in HRQoL and Associated Risk Factors in Colorectal Cancer Survivors: With a Focus on Social Determinants of Health (SDOH)

**DOI:** 10.1007/s12029-024-01070-2

**Published:** 2024-05-31

**Authors:** Claire J. Han, Fode Tounkara, Matthew F. Kalady, Anne M. Noonan, Electra D. Paskett, Diane Von Ah

**Affiliations:** 1 Center for Healthy Aging, Self-Management and Complex Care, Ohio State University College of Nursing, Ohio State University– James: Cancer Treatment and Research Center, Columbus, OH 43210 USA; 2 Department of Biomedical Informatics, Ohio State University College of Medicine, Ohio State University– James: Cancer Treatment and Research Center, Columbus, OH 43210 USA; 3 Division of Colon and Rectal Surgery, Clinical Cancer Genetics Program, Ohio State University– James: Cancer Treatment and Research Center, Columbus, OH 43210 USA; 4 GI Medical Oncology Selection, GI Oncology Disease Specific Research Group Leader, Ohio State University– James: Cancer Treatment and Research Center, Columbus, OH 43210 USA; 5 Department of Internal Medicine in the, College of Medicine, Division of Epidemiology, College of Public Health, Ohio State University, Columbus, OH 43210 USA; 6 Center for Healthy Aging, Self-Management, and Complex Care, Ohio State University, College of Nursing, Cancer Survivorship and Control Group, Ohio State University, Ohio State University– James: Cancer Treatment and Research Center, Columbus, OH 43210 USA

**Keywords:** Colorectal Cancer, Health-Related Quality of Life, Healthy Equity, Social Determinants of Health, Cancer Survivorship

## Abstract

**Purpose:**

This study aimed to understand how health-related quality of life (HRQoL) differs by race/ethnicity in colorectal (CRC) survivors. We aimed to 1) examine racial/ethnic disparities in HRQoL, and 2) explore the roles of social determinants of health (SDOH) risk factors for HRQoL differ by racial/ethnic groups.

**Methods:**

In 2,492 adult CRC survivors using Behavioral Risk Factor Surveillance System (BRFSS) survey data (from 2014 to 2021, excluding 2015 due to the absence of CRC data), we used the Centers for Disease Control and Prevention (CDC) HRQoL measure, categorized into “better” and “poor.” Multivariate logistic regressions with prevalence risk (PR) were employed for our primary analyses.

**Results:**

Compared with non-Hispanic Whites (NHW), non-Hispanic Blacks (NHB) (PR = 0.61, p = .045) and Hispanics (PR = 0.32, p < .001) reported worse HRQoL in adjusted models. In adjusted models, unemployed/retired and low-income levels were common risk factors for worse HRQoL across all comparison groups (NHW, NHB, non-Hispanic other races, and Hispanics). Other SDOH associated with worse HRQoL include divorced/widowed/never married marital status (non-Hispanic other races and Hispanics), living in rural areas (NHW and NHB), and low education levels (NHB and Hispanics). Marital status, education, and employment status significantly interacted with race/ethnicity, with the strongest interaction between Hispanics and education (PR = 2.45, p = .045) in adjusted models.

**Conclusion:**

These findings highlight the need for culturally tailored interventions targeting modifiable factors (e.g., social and financial supports, health literacy), specifically for socially vulnerable CRC survivors, to address the disparities in HRQoL among different racial/ethnic groups.

**Supplementary Information:**

The online version contains supplementary material available at 10.1007/s12029-024-01070-2.

## Introduction

Colorectal cancer (CRC) is the second leading cause of cancer-related deaths in the United States (U.S.), which are projected to cause approximately 170,968 new cases (+17.3% increase compared to the total number of cases in 2020) and 64,553 deaths by 2030 (+22.2% increase from 2020) [1]. While CRC affects various demographic groups, minority (i.e., racially and ethnically underrepresented) populations face a disproportionate burden, with non-Hispanic Blacks (NHB) experiencing the highest incidence and mortality rates at a 40% increased risk of death compared to non-Hispanic White patients (NHW) [1]. In addition to the racial and ethnic disparities observed in CRC screening rates, incidence, and mortality rates, studies have highlighted the complexity of these disparities, affecting not only incidence and mortality but also the quality of life and survivorship care across the entire CRC care continuum for CRC survivors in the U.S. [2].

These racial and ethnic cancer inequities in CRC survivorship are driven by a combination of factors, including differences in social determinants of health (SDOH), such as individual-level factors (e.g., socioeconomic status, education, healthcare access), and population-level factors i.e., structural racism (e.g., neighborhood and environment, social policy) [3]. In the U.S., racial/ethnic disparities in health are deeply intertwined with SDOH factors, resulting in privileges for individuals identified as White and disadvantages primarily affecting those identified as Black/African American, Asian, Native American, or individuals of Hispanic/Latinx ethnicity [3]. For example, NHB CRC survivors were more likely to have poorer SDOH status. These SDOH inequalities resulted in disparities in healthcare access to receive cancer treatment and survival care follow-up compared with non-Hispanic white CRC survivors [4, 5].

The health-related quality of life (HRQoL) encompasses physical and emotional well-being, influenced by health status and disease effects [6]. Cancer survivors encounter numerous challenges and potential long-term effects from their illness and treatments. HRQoL serves as a crucial measure of cancer survivorship, offering valuable insights into overall well-being, experiences following cancer treatment, and predicting cancer survival [6]. To our knowledge, five studies have explored racial and ethnic disparities in HRQoL among cancer survivors, including those with CRC [6–10]. Notably, NHB individuals had poorer HRQoL than NHW counterparts in two studies (CRC, 9% of 89 total subjects [6]; 13% of 232 total subjects [7]). Smithson et al. [8] also examined the racial and ethnic differences in HRQoL in 304 older adult CRC survivors and reported that NHB patients reported poorer HRQoL than NHW patients. Yost et al. [9] reported that the Hispanic race was associated with poor HRQoL in 496 CRC survivors compared to NHW. Overall, in the prior studies aforementioned mentioned above [6–10], CRC survivors are underrepresented in terms of understanding racial and ethnic disparities in HRQoL with small sample size or limited to older adults, and the primary drivers of worse HRQoL, which may vary among different racial and ethnic groups of CRC survivors, are unknown. Only two studies examined risk factors for HRQoL in 1,132 and 99 CRC survivors, respectively [10, 11]. Unmarried status and lower education levels were associated with poor HRQoL [10, 11]. However, these studies [10, 11] examined age, sex, marital status, education, alcohol consumption, and tobacco use associated with HRQoL but did not include broader aspects of the SDOH (e.g., living areas, economic status, health care access) based on the Healthy People 2030 SDOH framework [12]. This limitation hinders a comprehensive understanding of how SDOH risk factors for HRQoL differ among racial and ethnic groups, and it is not known which particular SDOH factors predominantly contribute to HRQoL disparities among cancer survivors from diverse racial and ethnic groups [11].

Understanding the specific or common risk factors for worse HRQoL across different racial.

and ethnic groups is crucial for supporting cancer survivorship and reducing health disparities. While existing research has focused primarily on racial/ethnic disparities in CRC incidence and mortality rates, investigations of HRQoL and potential SDOH risk factors for HRQoL, have been limited in large samples of CRC survivors. To address this gap, we used data from the Behavioral Risk Factor Surveillance System (BRFSS), a large population-based database, to examine 1) racial and ethnic differences in HRQoL and 2) the roles of SDOH in racial and ethnic differences in HRQoL. Among SDOH available in the BRFSS data, we specifically included individual-level SDOH based on the Healthy People 2030 SDOH framework (social and community context – marital status, living areas, education, economic stability, and health care access) [12]. We hypothesize that racial/ethnic differences will exist in HRQoL (Aim 1), and several SDOH will be associated with HRQoL, and this relationship may vary among different racial/ethnic groups (Aim 2). This approach allows us to determine whether the impact of selected SDOH risk factors on HRQoL varies among different racial/ethnic groups. This study focused on multiethnic CRC survivors, encompassing NHW, NHB, non-Hispanic individuals of other races (including American Indians and Alaskan Natives, Asians, Native Hawaiians/other Pacific Islanders), and Hispanics.

## Materials and Methods

### Data Source and Study Population

The data source for this study was the BRFSS, a nationwide telephone survey initiated by the Centers for Disease and Prevention (CDC) in 1984 [13]. BRFSS gathers information on health-related behaviors, demographic factors, SDOH, preventable causes of death, and preventive health practices among noninstitutionalized residents aged 18 or older in all 50 U.S. states, the District of Columbia, and three U.S. territories [13]. The survey used random digit dialing sampling covering landlines and cellular telephones. The validity and reliability of the BRFSS data have been demonstrated [13]. The study conducted a secondary data analysis using publicly available BRFSS survey data for CRC survivors from 2014 to 2021, excluding 2015 due to the absence of CRC data. The current cross-sectional study included adult CRC survivors over the age of 18 who had a history of CRC diagnosis from their medical providers. Individuals who refused to respond to survey questions or had missing responses for the included variables were excluded from the study. The original BRFSS study obtained approval from the Institutional Review Board (IRB). The current study was waived for IRB approval as a secondary data analysis.

### HRQoL: Primary Outcomes of Interest

The study utilized the CDC HRQoL measure, including self-reported general health status assessments. The primary outcome of interest was the general HRQoL. Participants were asked to rate their health as excellent, very good, good, fair, or poor for general health. The primary outcomes were categorized based on the validated cutoff points established by the CDC. General health was dichotomized into "better" for excellent, very good, or good responses and "poor" for fair or poor responses [13].

### Social Determinants of Health (SDOH): Potential Risk Factors of HRQoL

In the BRFSS data, we included SDOH factors, including economic status, education, social and community context, and healthcare access, based on the CDC Healthy People 2030 SDOH framework [12]. None of the variables related to quality of care or environmental factors (e.g., zip code, environmental safety, or transportation) were available in the BRFSS dataset of CRC survivors.

### Statistical Analysis

The BRFSS is strategically designed to collect comprehensive health-related data from the adult population across various U.S. states. To ensure the representativeness of the sample data, the BRFSS employs data weighting, encompassing population characteristics and survey design elements. Weighting involves two integral components: 1) design weight or factors which influence selection probability while accounting for nonresponse bias and noncoverage errors, and 2) adjustments to population demographics using methods such as ranking or interactive proportional fitting [14]. The present study adopted sophisticated survey procedures involving appropriate data stratification and weighting for the study sample.

We used a combination of descriptive statistics, bivariate analyses, and logistic regressions. Descriptive statistics were used to summarize the primary outcomes and participants’ characteristics. First, the chi-square or analysis of variance (ANOVA) test was used to explore the differences in HRQoL and SDOH by racial/ethnic groups. The current study is an exploratory study, thus, we did not perform the Bonferroni correction to reduce the risk of Type II errors (false negative). Second, we conducted logistic regressions to examine 1) the impact of racial/ethnic groups on HRQoL and 2) the roles of SDOH in racial/ethnic disparities in HRQoL. Univariate and multivariate logistic regressions were employed to compute unadjusted and adjusted prevalence ratios (PRs: known as risk ratios) and corresponding 95% confidence intervals (CIs). According to bivariate analyses, demographic and clinical characteristics were integrated into the regression models as covariates only if they exhibited significant associations with HRQoL. The final regression models also included adjustments for survey years as covariates to mitigate potential confounding effects. Correlation analyses, including SDOH, demographic, and clinical characteristics, were performed to evaluate the variance inflation factors (VIFs) to address multicollinearity. Variables with a VIF exceeding 5 indicated substantial multicollinearity [15, 16]. The mitigation of collinearity and model refinement were facilitated through stepwise eliminations in multivariate regression models [15, 16]. The average VIF value for our final model was 1.76, which is below the ‘standard’ 2.5 threshold, suggesting no detrimental multicollinearity in our model [15, 16].

To address our second aim, we first conducted subgroup analyses to present the results of the unadjusted and adjusted associations between SDOH and HRQoL separately by each racial/ethnic group. This allows for a detailed examination to identify primary SDOH risk factors for HRQoL within specific racial/ethnic groups [17]. Then, we further tested the interactions between ‘racial/ethnic groups’ and ‘selected SDOH associated with HRQoL in the subgroup analyses.’ This entails examining whether the associations between the selected SDOH and HRQoL differ significantly between racial/ethnic groups. This interaction analysis evaluates if the strength or direction of the association varies across different levels of the modifying variable, in this case, racial/ethnic groups [18]. Statistical analyses were conducted using R software. Unless otherwise specified, we used a threshold of p < 0.05 (two-sided) to determine statistical significance for all analyses.

## Results

### Participants’ Characteristics and Racial/Ethnic Differences in HRQoL

The study included an unweighted cohort of 2,492 colorectal cancer (CRC) survivors (Table 1). Upon extrapolating to state populations, these survivors represented 165,876 adults within the collective dataset from 2014 to 2021, except for 2015 (Table 1A). The majority of the respondents were White CRC survivors (82.1%), followed by Black CRC survivors (7.1%), other races (6.7%), and Hispanic CRC survivors (4.2%). The median age varied across the groups. The white group had the highest median age at 59.4 years, while the non-Hispanic other races had the lowest median age at 55.6 years (p = .051). A greater proportion of NHW (71.6%) were 65 years or older than were the other groups. The highest proportion of Hispanic individuals were aged 18–64 years (48.1%) (p < .001). The difference in sex distribution across the groups was not statistically significant, with a p-value of 0.457 (Table 1A). Over half of the CRC survivors reported a good HRQoL across racial/ethnic groups (Table 1A). While NHW reported the highest proportion of patients with a better HRQoL (67.2%), NHB had the highest prevalence of patients with a worse HRQoL (47.1%), followed by Hispanics (44.8%) (Table 1A).

**Table 1:** Participants’ Characteristics and HRQoL among CRC Survivors (Total Weighted Study N = 165,876; Unweighted N = 2,492)

**A. Participants’ characteristics and HRQoL**						
N (%) otherwise specified		**Non-Hispanics**			**Hispanics**	***F, p-value***^**a,b**^
		**White n = 2,045 (82.1%)**	**Black n = 176 (7.1%)**	**Other Races n = 167 (6.7%)**	**n = 104 (4.2%)**	
**Baseline characteristics**						
mean Age		58.2	56.8	56.4	54.9	4.576, .051
median (IQR)		59.4 [45, 98]	57.2 [44, 97]	57.9 [42, 98]	55.6 [38, 92]	
Age group						
18–64		562 (27.3)	71 (40.3)	70 (41.9)	50 (48.1)	46.3, < .001
65 or older		1468 (71.6)	105 (59.7)	97 (57.5)	54 (50.0)	
Sex						
Male		896 (43.7)	77 (43.8)	78 (46.7)	53 (51.0)	2.6, .457
Female		1154 (56.3)	99 (56.3)	89 (53.3)	51 (49.0)	
**General HRQoL**						15.1, .002
Better		1378 (67.2)	93 (52.9)	108 (64.8)	57 (55.2)	
Worse		672 (32.8)	83 (47.1)	59 (35.2)	47 (44.8)	

**B. Associations between Race/Ethnic Groups and HRQoL**						
**Better HRQoL (vs. Worse)**	**Unadjusted models**			**Adjusted models**^**d**^		
	PR	95% CI	*p-value*^c^	Adjusted PR	95% CI	*p-value*^c,d^
Non-Hispanic Blacks	0.78	0.68, 0.91	**< .001**	0.61	0.07, 0.92	.045
Non-Hispanic other races	0.98	0.79, 1.55	0.533	0.95	0.71, 1.42	.976
Hispanics	0.28	0.12, 0.77	**0.003**	0.32	0.11, 0.83	**< .001**

*IQR* Interquartile range

^a^p-values based on Chi-square or ANOVA test

^b^Significant findings (p < .05) were highlighted in bold

^c.^p-values based on multivariate logistic regression analyses with reference group as non-Hispanic Whites. Significant findings (p < .05) were highlighted in bold. Survey years were adjusted for both univariate and multivariate analyses

^d^For multivariate analyses, we controlled age group, pain, health risk behaviors, and comorbidities (see Supplementary Table) and survey years

To examine the impact of race and ethnic group on HRQoL (Table 1B), the PRs and adjusted PRs (APRs) and 95% CIs for each racial and ethnic group compared to the reference group (NHW) were calculated via unadjusted analyses. Compared to NHW, NHB had 0.78 times lower PR of having a better HRQOL (p < .001), and Hispanics had 0.28 times lower PR of having a better HRQoL (p = .003). We first conducted a chi-square or ANOVA test for multivariate analyses to identify potential covariates, such as baseline data, including SDOH related to HRQoL (Supplementary Table). After adjustment for covariates encompassing age group, pain, health risk behaviors, comorbidities, and survey years in multivariate logistic models (Table 1B), the NHB CRC survivors were 39% (APR = 0.61) less likely to have a better HRQoL than the NHW individuals were (p = .045). However, this significant difference decreased in the adjusted analysis for individuals of non-Hispanic other races and Hispanics (Table 1B).

### Racial/Ethnic Differences in SDOH Risk Factors for HRQoL

We found that the SDOH of CRC survivors varied significantly according to race and ethnicity, with all differences being statistically significant (p < .05), except for medical cost concerns (Table 2). Among the findings, NHW had the highest prevalence rates in several categories, including being married or partnered (49.9%), having a graduate or higher degree (29.4%), being employed (25.0%), or being retired (58.9%), having an income ranging from $50,000 to < $100,000 (annual household income), owning a home (82.7%), and having health insurance (95.6%). In contrast, a lower prevalence of annual household income of less than $25,000 was observed in the NHW than in the NHB and Hispanics (p < .001). NHB and Hispanic CRC survivors are more likely to be never married than are CRC survivors of NHW and other non-Hispanic races. Hispanic individuals, in particular, exhibited distinct attributes, including a high prevalence of having less than a high school education (22.1%) and unemployment (33.6%) among various racial/ethnic cohorts.

**Table 2:** Racial/Ethnic Differences in SDOH among CRC Survivors (Total Weighted Study N = 165,876; Unweighted N = 2,492)

N (%) otherwise specified	**Non-Hispanics**			**Hispanics** **n = 104 (4.2%)**	***F, p-value***^**a,b**^
	**White** **n = 2,045 (82.1%)**	**Black** **n = 176 (7.1%)**	**Other Races** **n = 167 (6.7%)**		
**SDOH: Social and Community Context**					
** Marital Status**					147.9, < **.001**
Married or partnered	1,020(49.9)	55 (31.3)	69 (41.3)	50 (48.1)	
Divorced, separated, or widowed	863 (42.2)	92 (52.3)	73 (43.7)	32 (30.7)	
Never married	162 (7.9)	29 (16.5)	19 (11.4)	13 (12.5)	
**Living in urban versus rural areas**					
Metropolitan	478 (39.9)	49 (56.0)	29 (38.6)	12 (40.0)	54.5, .**011**
Suburban	184 (15.4)	20 (22.0)	5 (5.3)	5 (16.7)	
Rural	536 (44.7)	20 (22.0)	42 (56.0)	13 (43.3)	
**SDOH: Education**					
Less than high school	132 (6.4)	25 (14.3)	25 (15.0)	23 (22.1)	59.9, < **.001**
High school	693 (33.9)	57 (32.4)	42 (25.1)	22 (21.2)	
College or technical school	611 (29.9)	48 (27.3)	52 (31.1)	31 (29.8)	
Graduate or higher	602 (29.4)	45 (25.6)	48 (28.7)	28 (26.9)	
**SDOH: Economic Stability**					
** Employment Status**					89.8, < **.001**
Employed	513 (25.0)	31 (17.6)	45 (27.0)	22 (21.2)	
Unemployed	322 (15.7)	49 (27.8)	38 (22.7)	35 (33.6)	
Retired	1,204(58.9)	95 (54.0)	83 (49.7)	45 (43.3)	
**Household Income/year**					67.6, < **.001**
Less than $25,000	514 (25.1)	70 (39.8)	57 (34.2)	30 (28.8)	
$25,000 to < $35,000	224 (11.0)	21 (11.9)	23 (13.8)	11(10.6)	
$35,000 to < $50,000	289 (14.1)	18 (10.2)	16 (9.6)	6 (5.8)	
$50,000 to < $100,000	621 (30.4)	36 (20.5)	41 (24.6)	26 (25.0)	
$100,000 or more	24 (1.2)	2 (1.1)	3 (1.8)	2 (1.9)	
**Homeownership**					125.3, < **.001**
Own	1,691(82.7)	104 (59.1)	110 (65.9)	66 (63.5)	
Rent	286 (14.0)	62 (35.2)	50 (29.9)	29 (27.9)	
**SDOH: Health Care Access**					
Health Insurance (Yes)	1,250(95.6)	99 (92.5)	87 (89.7)	49 (92.5)	27.9, .**001**
Time since last checkup with primary care providers (PCPs)					10.3, **.016**
within past year	1,767(86.4)	162 (92.0)	135 (80.8)	86 (82.7)	
more than the past year	278 (13.6)	14 (8.0)	32 (19.2)	18 (17.3)	
Medical cost concerns for health care access (Yes)	157(7.7)	16 (9.1)	12 (7.2)	10 (9.6)	6.3, .898

*SDOH* Social Determinants of Health

^a^p-values based on Chi-square or ANOVA test

^b^Significant findings (p < .05) were highlighted in bold

### Associations of SDOH (risk factors) with HRQoL differ by Racial/Ethnic Groups

#### Race/Ethnicity Subgroup Analyses

In our initial analyses to explore the associations between SDOH and HRQoL within different race/ethnicity categories, univariate and multivariate logistic regression models were conducted to investigate the most potent SDOH risk factors for HRQoL in each racial and ethnic group. Both unadjusted and adjusted PRs of potential risk factors for HRQoL according to racial/ethnic group are presented in Table 3AB (A. unadjusted; B. adjusted results). For unadjusted models, unemployed/retired status and low-income level (medial annual household income < $50 K) were significantly associated with worse HRQoL across all racial and ethnic groups. In the social and community context, divorced/widowed/never married status was associated with worse HRQoL in non-Hispanic other races (APR = 1.09, p = .046) and Hispanics (APR = 1.05, p = .048). CRC survivors living in rural areas reported worse HRQoL in NHW (APR = 1.41, p = .021) and NHB (APR = 1.99, p = .035) than in metropolitan or suburban areas. CRC survivors with low education levels had a 29% and 17% increase in the prevalence of worse HRQoL in NHB and Hispanics, respectively, compared to those with high education levels in these groups. Similar results were found in adjusted multivariate models. In adjusted models, annual household income was the most significant SDOH risk factor for HRQoL in NHW (APR = 1.48, p = .005), and non-Hispanic other races (APR = 1.25, p = .048), while employment status was the most significant SDOH risk factor for HRQoL in NHB (APR = 1.56, p = .001) and Hispanics (APR = 1.89, p = .014).

**Table 3:** The Roles of SDOH in Racial/Ethnic Disparities in HRQoL

**A. Data presented as unadjusted PR, 95% CI & p-value**				
**Potential Risk Factors of HRQoL** **(Worse versus Better)**	**Non-Hispanics**			**Hispanics**
	**White**	**Black**	**Other Races**	
**Social and Community Context**				
Marital Status (Divorced/widowed/never married). Ref. Married or partnered.	0.96 (0.87, 1.56), .964	0.59 (0.21, 1.89), .652	1.09 (1.01, 1.42), **.046**	1.05 (1.01, 1.42), .**048**
Living in a rural area. Ref. Living in metropolitan/suburban.	1.41 (1.25, 1.91), .**021**	1.99 (1.54, 2.41), .**035**	1.33 (0.55, 3.12), .891	1.23 (0.94, 1.59), .901
Living in suburban/rural. Ref. Living in metropolitan.	1.05 (0.74, 1.99), .435	1.43 (0.12, 2.53), .451	2.41 (0.98, 4.91), .984	1.53 (0.81, 2.19), .742
**Education**				
High school or less. Ref. College/graduate or higher	0..99 (0.42, 1.78), .949	1.29 (1.01, 2.33), .**011**	1.35 (0.89, 1.33), .549	1.17 (1.01, 1.35), **.029**
**Economic Stability**				
Employment Status (Unemployed/Retired). Ref. Employed	1.33 (1.07, 1.79), **.049**	1.78 (1.11, 2.13), **.007**	1.17 (1.01, 1.78), **.004**	1.54 (1.13, 2.87), **.035**
Household Income/year categorized by a median of < $50 K. Ref. ≥ $50 K.	1.69 (1.03, 1.90), **.018**	1.58 (1.31, 2.03), .**045**	1.55 (1.28, 2.31), .**032**	1.90 (1.32, 2.59), .**003**
Homeownership. Rent. Ref. Own	0.98 (0.93, 1.44), .581	1.01 (0.99, 1.87), .892	0.98 (0.35, 2.88), .894	1.32 (0.89, 1.89), 884
**Health Care Access**				
Health insurance Rent. Ref. Own	1.43 (1.18, 1.93), .184	1.95 (0.85, 3.41), .766	1.33 (0.54, 2.22), .841	0.87 (0.31, 1.43), .952
Time since last checkup with primary care providers (PCPs)				
More than the past year. Ref. Within past year.	1.22 (0.19, 1.94), .512	0.99 (0.43, 1.43), .312	2.31 (0.33, 4.51), .822	1.41 (0.43, 2.09), .212
Medical Cost Concerns (Yes) Ref. No concerns	1.35 (0.75, 2.13), .065	0.94 (0.33, 2.84), .542	1.03 (0.45, 3.87), .980	1.19 (0.87, 1.89), .999

**B. Data presented as adjusted PR (APR), 95% CI & p-value**^**a**^				
**Social and Community Context**				
Marital Status (Divorced/widowed/never married). Ref. Married or partnered.	Not applicable	Not applicable	1.35 (1.03, 2.41), **.001**	1.15 (1.01, 1.98), **.016**
Ref. Living in rural. Living in metropolitan/suburban.	1.11 (1.05, 1.94), .**035**	1.35 (1.54, 2.22), .**043**	1.11 (0.05, 2.75, .999	0.95 (0.55, 1.65), .905
**Education**				
Ref. Education (High school or less). College/graduate or higher	Not applicable	1.11 (1.03, 1.54), **.035**	Not applicable	1.32 (1.12, 1.63), **.001**
**Economic Stability**				
Employment Status (Unemployed/Retired). Ref. Employed	1.15(1.09, 1.66), **.033**	1.56 (1.15, 2.13), **.001**	1.17 (1.04, 2.63), < **.001**	1.89 (1.31, 3.12), .**014**
Household Income/year categorized by a median of < $50 K. Ref. ≥ $50 K.	1.48 (1.19, 1.73), **.005**	1.32 (1.01, 2.34), .**045**	1.25 (1.18, 1.94), .**048**	1.53 (1.12, 1.90), .**019**

Significant findings (p < .05) were highlighted in bold

The survey years of the BRFSS dataset were controlled for both unadjusted and adjusted models

For multivariate analyses, we also controlled age group, pain, health risk behaviors, and comorbidities (see Supplementary Table), and SDOH showing significant associations with HRQoL in each group

^a^APRs were based on stepwise multivariate logistic regression models

#### Interactions Between SDOH and Racial/Ethnic Groups

Given the results above, which show that the PRs of SDOH for the HRQoL differ significantly by racial/ethnic groups, it suggests that race/ethnicity moderates the relationships between SDOH and HRQoL in our study. Therefore, to investigate the underlying mechanisms of racial/ethnic differences in HRQoL further, we then tested the interactions between race and selected SDOH factors (Table 4). The selected SDOH included marital status, living areas, education, employment status, and annual household income, demonstrating significant relationships with HRQoL in our prior multivariate analyses (Table 3B). In assessing the interaction between race/ethnicity and the selected SDOH impacting on HRQoL, the results of crude and adjusted PRs (APRs) were similar (Table 4). In groups with divorced, widowed, or never-married marital status, non-Hispanic individuals of other races (APR = 1.45; 95% CI: 1.17–2.48, p = .014) and Hispanic CRC survivors (APR = 1.11; 95% CI: 1.04–1.99, p = .035) reported had a higher prevalence of worse HRQoL, while NHB individuals had 11% decrease in prevalence of worse HRQoL (APR = 0.89; 95% CI: 0.24–0.71, p < .001) compared to NHW individuals. Among groups with low education levels, NHB (APR = 1.77; 95% CI: 1.35–2.34, p = .012) and Hispanics (APR = 2.45; 95% CI: 1.33–3.14, p = .045) had a higher risk of worse HRQoL than NHW. Unemployed or retired CRC survivors had 1.65 and 1.76 times the prevalence of worse HRQoL in NHB, and Hispanics, respectively, compared to unemployed or retired NWH. Annual household income levels and living areas did not show any significant interactions with race/ethnicity.

**Table 4:** Interactions between SDOH and Racial/Ethnic Groups

**Worse versus Better HRQoL (Ref)**	**Crude**^**a**^** PR [95% CI]**^**c**^	**Adjusted PR**^**a,b**^** (APR) [95% CI]**^**c**^
**Race/Ethnicity x Marital Status Interaction (if divorced/widowed/never married = 1, married/partnered = ref.)**		
Non-Hispanic Whites (ref)	1.00	1.00
Non-Hispanic Blacks	0.81 [0.22 -0.99],** p < .001**	0.89 [0.24 - 0.99], **p = .004**
Non-Hispanic Other Races	1.55 [1.07–2.68], **p < .001**	1.45 [1.17–2.48], **p = .014**
Hispanics	1.11 [1.03–2.09], **p = .012**	1.11 [1.04–1.99],** p = .035**
**Race/Ethnicity x Living Areas Interaction (if rural = 1, metropolitan/suburban = ref.)**		
Non-Hispanic Whites (ref)	1.00	1.00
Non-Hispanic Blacks	1.11 [0.23 - 1.43]. p = .422	1.98 [0.75–2.41]. p = .982
Non-Hispanic Other Races	0.91 [0.42 - 2.49], p = .984	1.33 [0.35 - 1.93], p = .522
Hispanics	1.35 [0.44 -1.98], p = .512	2.01 [0.91 - 3.04]. p = .841
**Race/Ethnicity x Education Interaction (if high school or less = 1, college/graduate or higher = ref.)**		
Non-Hispanic Whites (ref)	1.00	1.00
Non-Hispanic Blacks	1.89 [1.12 - 2.78], **p < .001**	1.77 [1.35 - 2.34], **p = .012**
Non-Hispanic Other Races	0.86 [0.56 - 0.94], **p = .035**	1.02 [0.56 - 1.32], p = .423
Hispanics	2.89 [1.55 - 3.41],** p = .013**	2.45 [1.33 - 3.14],** p = .045**
**Race/Ethnicity x Employment status Interaction (if unemployed/retired = 1, employed = ref.)**		
Non-Hispanic Whites (ref)	1.00	1.00
Non-Hispanic Blacks	1.85 [1.22 - 3.91], **p = .031**	**1.65 [1.12 - 2.01], p < .001**
Non-Hispanic Other Races	1.12 [0.65 - 1.32], p = .059	1.51 [0.84 - 2.41], p = .176
Hispanics	1.94 [1.24 - 2.56], **p = .042**	**1.76 [1.07 - 2.55], p = .043**
**Race/Ethnicity x Income Interaction (if household income based on the median, less than $50 K/year = 1, higher than $50 K/year = ref.)**		
Non-Hispanic Whites (ref)	1.00	1.00
Non-Hispanic Blacks	1.01 [0.42–1.55], p = .521	0.99 [0.56–1.42], p = .451
Non-Hispanic Other Races	1.44 [0.13–2.51], p = .412	1.31 [0.12 -2.41], p = .523
Hispanics	1.35 [0.56 -1.51], p = .951	1.09 [0.43–1.33], p = .895

^a^The survey years of the BRFSS dataset were controlled for both crude PR and APR analyses

^b^APRs were based on stepwise multivariate logistic regression models. For multivariate analyses including interaction terms, we also controlled age group, pain, health risk behaviors, and comorbidities (see Supplementary Table), and SDOH showing significant associations with HRQoL in each group

^c^Significant findings (p < .05) were highlighted in bold

## Discussion

To date, racial and ethnic disparities in the risk and mortality of CRC have been emphasized [2], whereas investigations of these disparities in CRC survivorship outcomes, specifically for HRQoL, are lacking. In addition to assessing racial and ethnic disparities in CRC risk and mortality, our study is the first to use large sample population data (BRFSS) to address racial/ethnic differences in HRQoL and to provide comprehensive insights into the multifaceted SDOH factors contributing to HRQoL disparities among different racial and ethnic groups of CRC survivors.

Our study highlights racial/ethnic disparities in HRQoL, particularly among NHB and Hispanic CRC survivors. These disparities are linked to unfavorable SDOH with worse HRQoL across different racial/ethnic groups. By identifying driving risk factors for HRQoL within each race/ethnic group, we underscore the need for targeted interventions for socially vulnerable CRC populations. Each race/ethnicity exhibits common (i.e., employment status, annual household income levels) or different (i.e., marital status, living areas, education levels) SDOH risk factors for HRQoL. In our final adjusted model, race/ethnicity and SDOH interactions, marital status, living areas, education levels, and employment status affect HRQoL differently across racial/ethnic groups. Notably, despite NHW CRC survivors reporting greater HRQoL than Hispanics in our study, even NHW CRC survivors may face HRQoL disparities with poor SDOH, warranting interventions for all races/ethnicities to improve their overall well-being. The differences in SDOH among racial/ethnic groups have been corroborated by previous studies of prostate cancer survivors [19]. For example, a meta-analysis of studies investigating prostate cancer outcomes among Black and White patients revealed that the interaction between race and SDOH is associated with racial disparities in prostate cancer outcomes, including increased mortality, poor healthy lifestyles, and greater comorbidities, in Black prostate cancer survivors compared with White prostate cancer survivors [19]. In a study of 8,582 individuals in the U.S. in the general population, similar results were found: poor SDOH was found in NHB and Hispanics compared to the NHW group [20]. Our findings can inform cancer community advocates and stakeholders about HRQoL disparities and SDOH-related root causes, advocating for policies, programs, and resources to provide targeted interventions for CRC survivorship.

Our study highlights the critical role of several SDOH**—**including marital status, education, and economic status—in shaping racial and ethnic disparities in HRQoL. Our findings suggest that social and cultural factors are associated with HRQoL amon**g** CRC survivors [21]. For example, family support linked to marital status appears crucial for the well-being of non-Hispanic other races and Hispanics. Asian/Pacific Islanders and Hispanic cultures prioritize interdependence and mutual support within the family, which could inform future interventions tailored to these population [22]. Furthermore, educational levels and employment status significantly contribute to racial/ethnic disparities, particularly among NHB and Hispanic individuals. Despite NHB and Hispanics being younger than NHW in our study, the impact of employment status on HRQoL is more pronounced in NHB and Hispanics. This finding suggests that economic inequalities cannot be solely explained by age differences; other factors, such as physical functioning, financial stability, and family caregiving responsibilities, may contribute to the higher prevalence of unemployed/retired status among NHB and Hispanics [23]. However, we should interpret the employment data with caution, as the economic status (e.g., income levels) between the unemployed and retired groups is unknown.

However, the interpretation of employment data should be cautious as the economic status (e.g., income levels) between unemployed/retired is unknown. Data from a previous study on cancer survivors [24] support our findings, indicating that having a job or returning to work after cancer treatments can be beneficial from societal engagement, rewards, and professional growth. These factors profoundly impact HRQoL, especially within racial and ethnic minority cancer populations [25–28]. In our study, the income variable did not exhibit interaction with race/ethnicity. This lack of interaction could be attributed to the limitations of our income variable, which focuses solely on annual household income. It may not fully encompass federal poverty levels, and other financial assets such as stocks, real estate, or pensions. Additionally, structural racism at the community levels (e.g., policy and area-level indices) may contribute to the observed inequalities in HRQoL. These disparities may arise from factors such as unequal access to education, transportation barriers at the community levels, residential segregation, exposure to discrimination and violence, living in unsafe neighborhoods, lack of job opportunities and working environments, structural policy and discrimination, and facing barriers to community healthcare access such as limited availability of gymnastic or exercise programs and inadequate environmental support [5, 29–31].

### Future Interventions and Clinical Implications

Our study underscores the necessity for future interventions targeting modifiable factors and bolstering education on self-management skills like coping, resiliency, and self-efficacy, alongside enhancing family and social support at the individual level. Government agencies and healthcare systems must offer improved support at the societal and institutional levels. Interventions should adopt a comprehensive approach, integrating healthcare delivery, social support networks, and community engagement initiatives. Implementing patient navigation programs can aid vulnerable cancer patients in surmounting care barriers and providing guidance on healthcare access, transportation, and financial resources. Additionally, integrating social services into clinical settings, such as on-site counseling and financial assistance, can ameliorate patient well-being and alleviate the burden of cancer-related physical and psychological symptoms. Collaborative efforts among healthcare providers, policymakers, community organizations, and advocacy groups are vital for successful intervention implementation, particularly for underserved populations [4, 32–34].

### Limitations

First, we used self-reported BRFSS data, and the findings may have recall bias. Second, we did not adjust for potential covariates of HRQoL, including clinical characteristics (e.g., psychological symptoms, cancer stages, types of cancer treatments received, time since cancer diagnosis), diet, and environmental factors (e.g., zip code, environmental safety, and transportation), because this information was not collected in the BFRSS, which will be related to selection bias. Third, in our study, our SDOH variables were limited to the individual-level SDOH rather than community-based (i.e., macro level) SDOH. Further examinations of the barriers at the social structural levels experienced by the racial/ethnic minority groups are warranted. We also did not apply the Bonferroni correction. Thus, there may be a potential for a Type I error (false positive). Finally, our study was underpowered for racial and ethnic minority groups compared to that of the NHW, with potential model overfitting. To minimize this limitation, we used PRs instead of odds ratios (ORs), and conducted subgroup analyses for each race/ethnic group to capture any unique patterns or associations between SDOH and HRQoL within each subgroup [35]. This suggests a future research direction, indicating that public surveys for cancer populations should be more inclusive of minority populations, including NHB, non-Hispanic other races, and Hispanic cancer survivors.

In conclusion, racial and ethnic disparities in HRQoL exist among CRC survivors, and these disparities intersect with SDOH. Addressing these disparities requires a multifaceted approach, specifically considering modifiable factors at both the individual and socio-community levels for the vulnerable CRC population.

### Supplementary Information

Below is the link to the electronic supplementary material.

Supplementary file1 (DOCX 19 KB)

## Data Availability

Behavioral risk factor surveillance system (BRFSS) survey data and questionnaires are publicly available to researchers on the BRFSS website. https://www.cdc.gov/brfss/annual_data/annual_data.htm.
