# Supplementary material for: Racial/Ethnic Disparities in HRQoL and Associated Risk Factors in Colorectal Cancer Survivors: With a Focus on Social Determinants of Health (SDOH)
Source: J Gastrointest Cancer. 2024 May 31;55(3):1179–89. doi: 10.1007/s12029-024-01070-2 (PMC11347476; doi:10.1007/s12029-024-01070-2)
Supplement: Supplementary file 1 — Supplementary file1 (DOCX 19 KB) [file 12029_2024_1070_MOESM1_ESM.docx]

Supplementary Table. Correlates with HRQoL (Bivariate analyses).

(Total Weighted Study N = 165,876; Unweighted N = 2,492)

| **% otherwise specified** | **Better HRQoL** | **Worse HRQoL** | **F, p-value** |
| --- | --- | --- | --- |
| **mean age (standard deviation)** | 58.8 (15.6) | 58.3 (16.1) | 141.6, .452 |
| Age group 18-64 | 27.1 | 35.8 | 20.7, <.001 |
| 65 or older | 71.9 | 63.0 |  |
| Sex Male | 44.4 | 43.4 | 3.24, .618 |
| Female | 55.6 | 56.6 |  |
| **Cancer Health Measures, Yes (versus No)** | | | |
| **Current physical pain from cancer** | 8.6 | 22.5 | 82.7, <.001 |
| **Health Risk Behaviors** | | | |
| Current smokers (Yes) versus  Former or never | 7.0 | 17.0 | 68.4, <.001 |
| Physically active participation | 73.6 | 50.5 | 134.7,<.001 |
| Heavy Alcohol Consumption | 65.2 | 34.8 | 4.908, .090 |
| **Comorbidities** | | | |
| Has chronic conditions >2 35.0 61.2 72.3, **<.031** | | | |

Note. Significant findings (p <.05) were highlighted in bold. Given the exploratory analyses, we did not adjust the Bonferroni correction.
